# Compensation for Retinal Vessel Density Reduces the Variation of Circumpapillary RNFL in Healthy Subjects

**DOI:** 10.1371/journal.pone.0120378

**Published:** 2015-03-18

**Authors:** Ivania Pereira, Stephanie Weber, Stephan Holzer, Georg Fischer, Clemens Vass, Hemma Resch

**Affiliations:** 1 Center for Medical Statistics Informatics and Intelligent Systems, Section for Medical Information Management and Imaging, Medical University Vienna, Vienna, Austria; 2 Department of Ophthalmology & Optometry, Medical University Vienna, Vienna, Austria; Medical University of South Carolina, UNITED STATES

## Abstract

This work intends to assess circumpapillary retinal vessel density (RVD) at a 3.46 mm diameter circle and correlate it with circumpapillary retinal nerve fiber layer (RNFL) thickness measured with Fourier-Domain Optical Coherence Tomography. Furthermore, it aims to evaluate the reduction of intersubject variability of RNFL when considering RVD as a source of information for RNFL distribution. For that, 106 healthy subjects underwent circumpapillary RNFL measurement. Using the scanning laser ophthalmoscope fundus image, thickness and position of retinal vessels were assessed and integrated in a 256-sector RVD profile. The relationship between local RVD value and local RNFL thickness was modeled by linear regression. RNFL was then compensated for RVD variation by regression formulas. A strong statistically significant intrasubject correlation was found for all subjects between RVD and RNFL profiles (mean R = 0.769). In the intersubject regression analysis, 247 of 256 RNFL sectors showed a statistically significant positive correlation with RVD (mean R = 0.423). RVD compensation of RNFL resulted in a relative reduction of up to 20% of the intersubject variance. In conclusion, RVD in a 3.46mm circle has a clinically relevant influence on the RNFL distribution. RVD may be used to develop more individualized normative values for RNFL measurement, which might improve early diagnosis of glaucoma.

## Introduction

Glaucoma is the second most frequent cause of irreversible blindness in industrialized nations. It is estimated that in 2020, about 80 million of people will be affected by the disease.[1] While there is currently no cure for this disease, treatment can delay its progression, and so early and accurate diagnosis of glaucoma is important to preserve vision.

Early glaucoma diagnosis is partially based on retinal nerve fiber layer (RNFL) thickness, which can be assessed by scanning laser polarimetry (SLP) or optical coherence tomography (OCT). RNFL becomes thinner in pathological cases, compared with healthy eyes,[2–8] due to the irreversible loss of the ganglion cells and their axons. However, the discrimination in RNFL thickness measurements between healthy and glaucoma cases is impaired by considerable interindividual variance among the healthy population. Some of the factors driving this variance can be compensated for, like sex, age and ethnicity, while others such as optic disc (OD) size and axial length [7–15] are presently not considered.

It has been shown by others [16,17] and by our group [18,19] that one additional source of interindividual variance of the RNFL is the variable circumpapillary distribution of the retinal blood vessels. A correlation was reported [16,17] between major temporal retinal blood vessels and major maxima of peripapillary RNFL thickness. Our group [18] reported a correlation between smaller angles of the major temporal vessels and a more oblique course of the nerve fiber bundles of the RNFL. Recently we introduced the circumpapillary retinal vessel density profile (RVD) as a function of distribution and thicknesses of the retinal vessels.[19] We have shown that the circumpapillary RVD as measured at the OD border may help to partially explain the RNFL variation.

Considering that during embryonic development, retinal vessels follow the paths and nutrient demands of the sprouting axons, it is presumable that measuring both the RVD and RNFL thickness at the same location might improve the previously reported associations. In the present work, we investigated the correlation between peripapillary RNFL thickness measured with Fourier-Domain OCT (FD-OCT) and RVD, both assessed at the same location (3.46 mm diameter circle).

## Subjects and Methods

### Subjects

This study was performed in collaboration between the Department of Ophthalmology and Optometry and the Section for Medical Information Management and Imaging of the Center for Medical Statistics Informatics and Intelligent Systems of the Medical University of Vienna. The protocol was approved by the Ethics Committee of the Medical University of Vienna, Austria, and written informed consent was obtained from all volunteers. The Guidelines of Good Clinical practice and the Declaration of Helsinki were followed.

Inclusion and exclusion criteria and examination procedures were described elsewhere.[19]

## Methods

The selection and measurement of the retinal vessels was performed using individual SLO images acquired with an OD centered protocol from Cirrus FD-OCT. The correspondent RNFL measurements were also used in this analysis. A customized software was developed in Matlab (Matlab R2012b, Mathworks Inc., Massachusetts, USA), similar to the one developed for the previous model.[19] For each image, a trained grader identified the OD margin and, from its center, three concentric circles were automatically placed on the image. One of those circles was the RNFL measurement circle defined by FD-OCT, with diameter of 3.46 mm. Two additional circles, with diameters 3.57 mm and 3.35 mm, were added for robustness of the measurements. Using these circles, the grader identified the borders of all measurable vessels visible in the SLO image and crossing the specified circles. The measured vessel thickness and the respective coordinates along the three circles were averaged, to obtain one thickness value and a center point for each vessel. For each vessel we determined the angle of the intersection between a horizontal line passing through the OD center and a line between OD center and the vessel center point. RVD profile for all subjects was obtained in a similar fashion as previously described [19] and optimized for median intersubject correlation maximization.

**Statistical analysis**. Statistical analysis was performed using the SPSS software package (SPSS Inc. USA) release No. 21.0.0.0. All statistical significant results present p<0.05. A statistical analysis similar to the first model including intra- and intersubject correlations was performed in order to allow a comparison between methods.[19]

A modified temporal-superior-nasal-inferior-temporal (TSNIT) graph was defined based on the slopes of the regression lines of all sectors, according to the formula *y* = *mx* + *b*. The slope is considered statistically significant if both limits of the 95% CI have an equal sign (either both positive or both negative).

Additionally, we calculated the difference between the 10^th^ and 90^th^ percentiles of RVD for each sector. This difference was multiplied with the according regression slope, to analyze the impact of vessel thickness on RNFL profile for subjects with more atypical RVD profile.

Furthermore, the RVD-compensated RNFL thickness was determined using equation (1) and applying it to each sector of each subject.

$$RNFL_{compensated}=RNFL_{measured}-\left(RVD_{subject}-RVD_{mean}\right)\times slope$$ (1)

The coefficients of variance (CV), both for the measured and the RVD-compensated RNFL thickness values, were calculated for each of the 256 sectors, as well as for the 12 clock hour sectors.

## Results

### Description of statistical subset

We used the same statistical subset, which has been described,[19] to allow direct comparison with the published results. The demographic distribution of the sample is provided in Table 1.

**Table 1:** Subjects demographic characteristics.

**Number of subjects**	106
**Sex (male/female)**	49/57
**Eye (right/left)**	66/40
**Age (mean years ± SD** ^**†**^ **)**	36.9±16.9
**Refractive Error (mean diopters ± SD** ^**†**^ **)**	0.05±1.46
**Mean Deviation (mean dB ± SD** ^**†**^ **)**	-0.13±1.04

^†^ SD = Standard Deviation

The parameters used for Gaussian-shaped convolution were N = 47, α = 2.7 for RVD measurements along the RNFL measurement area.

### Correlation between RVD and RNFL

To better understand the sources of the variability of RNFL measurements in healthy subjects, we analyzed how RVD was correlated with RNFL profile at a 3.46 mm diameter circle. Results showed that all individual RVD measurements (106 subjects) have a statistically significant, mostly strong positive correlation with the respective RNFL profile (mean value 0.769 ± 0.117). Moreover, intersubject linear regression analysis resulted in a significant Pearson correlation coefficient in 96% of the sectors (mean value of 0.423 ± 0.121). Comparing to the previous model, the significant sectors increased from 181 to 247 (out of 256) sectors, and the mean intersubject correlation coefficient increased by 62% (from 0.260 to 0.423). Detailed results are presented in Table 2. Values between brackets refer to the previous model.

**Table 2:** Statistical results for intra- and intersubject Pearson correlation coefficients analysis: comparison to the previous model.

	Intrasubject correlation coefficients: New model (old model)	Intersubject correlation coefficients: New model (old model)
**significance**	106 subjects (101)	247 sectors (181)
**max.**	0.955 (0.839)	0. 652 (0.490)
**min.**	0.314 (0.150)	0.083 (0.000)
**mean**	0.769 (0.535)	0.423 (0.260)
**S.D.**	0.117 (0.164)	0.121 (0.114)
**25 percentile**	0.705 (0.418)	0.344 (0.176)
**75 percentile**	0.843 (0.674)	0.505 (0.348)
**90 percentile**	0.899 (0.744)	0.596 (0.405)

**Fig 1:**
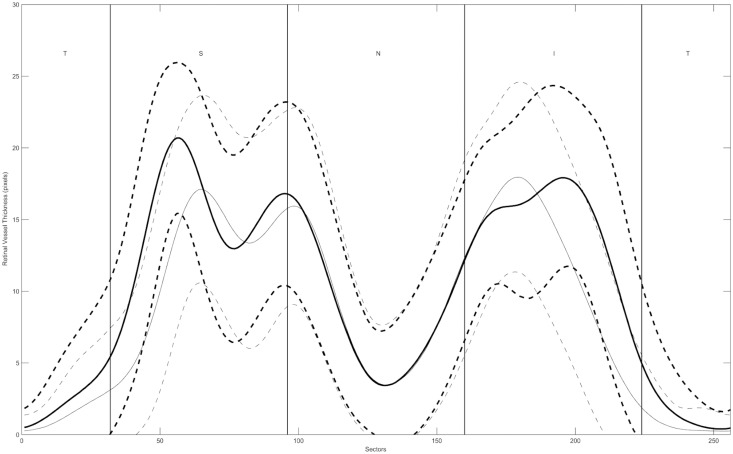
Average and Standard deviation of Retinal Vessel Density. Temporal—superior—nasal—inferior—temporal (TSNIT) graph for the average measurements of retinal vessel density (RVD) distribution in 106 healthy subjects. Solid lines represent the mean value and dashed lines represent average ± 1SD. Thicker lines refer to RVD profiles determined in a 3.46 mm diameter circle and thinner lines refer to RVD at the optic disc margin.

The average RVD profiles for both models are illustrated in Fig. 1 (thicker lines refer to the present model; thinner lines refer to the previous model). The double hump pattern of the RVD distribution resembles the shape of the RNFL distribution for both models. However, the temporal-superior and the temporal-inferior peaks of RVD are shifted between 10° and 15° in the temporal direction when RVD was measured at 3.46 mm (new model) as compared to measurement at the OD margin (old model).

### Regression analysis between RVD and RNFL

**Fig 2:**
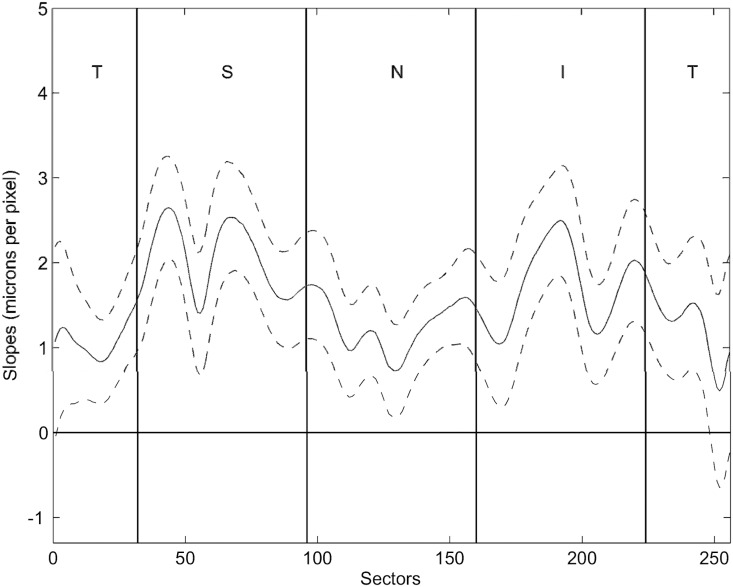
Modified TSNIT graphs of the regression coefficients. Modified Temporal—superior—nasal—inferior—temporal (TSNIT) graph plotting the regression coefficients obtained from the regression analysis (solid line) and the respective 95% confidence interval (CI) (dashed lines).

A modified TSNIT profile (Fig. 2), based on the intersubject linear regression analysis, displays the regression slopes of the 256 sectors. The solid line represents the intersubject regression slopes for each sector and the dashed lines refer to the 95% CI of the slopes.

The slopes were significantly positive for almost every sector (i.e. the 95% CI did not include the zero line), with the exception of a part of the papillo-macular bundle area. Maxima values were reached in the superior-temporal region. Comparing with our previous model [19], there was a pronounced increase in slopes in large parts of the TSNIT profile obtained for the 3.46 mm measurement circle. The average slope increased from 1.05 to 1.55 µm/pixel in the new model.

### Influence of atypical RVD in RNFL distributions

**Fig 3:**
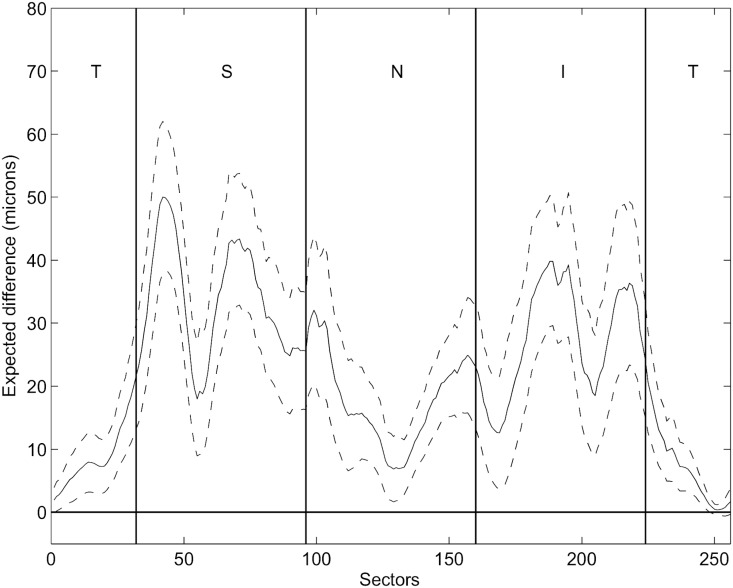
Estimated impact of RVD on RNFL thickness. Estimated impact of retinal vessel density (RVD) variation on retinal nerve fiber layer (RNFL) thickness. Solid line represent the difference between the 10% most extreme values of RVD, multiplied by the regression coefficients; dashed lines refer to the 95% CI.

To determine the potential clinical relevance of our results, we estimated the impact of RVD variation on RNFL thickness for cases with more atypical RVD. This was achieved by calculating the difference between the 10^th^ and 90^th^ percentiles of the RVD and multiplying these values by the respective intersubject regression slopes (Fig. 3). Regions with higher differences concentrate in superior and inferior regions, with a maximum difference up to approximately 50 microns.

### Reduced RNFL variance by compensation for RVD variation

**Fig 4:**
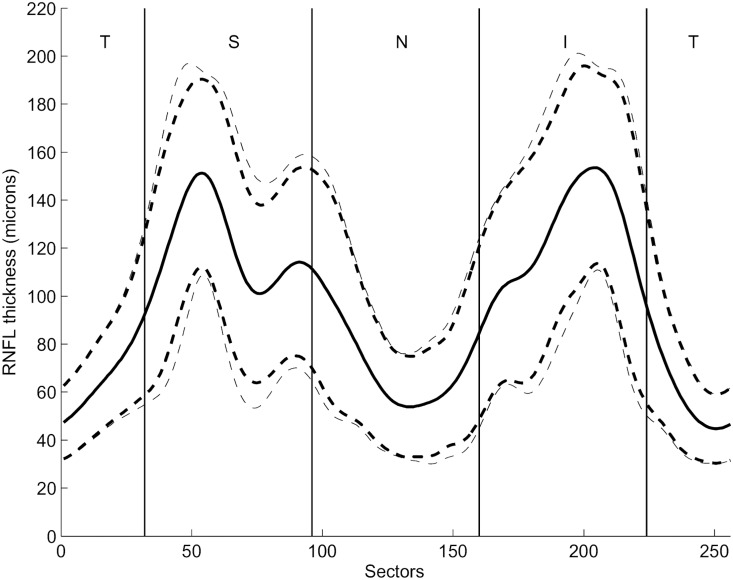
Profile of the RVD-compensated model of RNFL thickness, calculated for 106 healthy subjects according to equation 1. Solid lines refer to average values of retinal nerve fiber layer (RNFL) thickness (measured and compensated model values) and dashed lines refer to mean ± 2 standard deviation (SD). Thinner lines represent the measured values while thicker lines represent the compensated model values.

Finally, we compensated the measured RNFL profiles for the impact of RVD variation. This was achieved using the regression formulas and equation (1) to normalize the measured RNFL thickness of each subject and sector to the expected value for a subject with average RVD of this sector. Fig. 4 shows the profile of the intersubject variability, both for the measured values of RNFL thickness and for the RVD-compensated model. The mean ± 2xSD values of the measured RNFL thickness (thinner lines) are plotted against the mean ± 2SD values of the RVD-compensated model (thicker lines). Mean values are not influenced by the compensation process, but the variation is considerably reduced by the compensation. On average, the CV for the measured RNFL thickness was 18%, while for the RVD-compensated model it was 16%, meaning a relative reduction of variance of 11%. The maximum difference was in the temporal superior area, where the CV for the measured RNFL thickness was 18.7, which was reduced by our compensation to 15%, a relative reduction of 20%. Further results are present in Table 3.

**Table 3:** Coefficients of Variance (CV) for retinal nerve fiber layer (RNFL) thickness measured by Fourier Domain Optical Coherence Tomography (FD-OCT) and model-compensated for retinal vessel density (RVD).

Sectors	CV RNFL (FD-OCT)	CV RNFL (model)	CV Difference
**Sector 1**	15.221	14,879	-0.342
**Sector 2**	16,258	15,053	-1,205
**Sector 3**	18,742	15,045	-3,696
**Sector 4**	17,346	15,083	-2,263
**Sector 5**	16,592	14,915	-1,677
**Sector 6**	19,796	18,339	-1,457
**Sector 7**	18,629	17,733	-0,896
**Sector 8**	20,852	18,016	-2,836
**Sector 9**	18,790	18,100	-0,690
**Sector 10**	17,570	14,807	-2,763
**Sector 11**	16,970	15,480	-1,491
**Sector 12**	19,177	17,711	-1,465

When comparing with our previous model, variance is much more reduced at the best location (20% instead of 11%). The maximum reduction still concentrates on the superior region, but the current results present now a more pronounced variance reduction across the entire RNFL profile.

## Discussion

In this paper, we report that the RNFL thickness in healthy volunteers has a strong intraindividual correlation with the RVD profile, which reflects retinal vessels thickness and distribution on a 3.46 mm diameter circle around the optic disc. Intersubject correlation between RNFL and RVD was moderate, with a mean correlation coefficient of 0.423. The RVD profile, on average, may explain 18%—for some regions, up to 43%—of the RNFL intersubject variance. This is a meaningful improvement compared to our older model,[19] where, on average, the RVD explained 7% (up to 24%) of the RNFL variation. Furthermore, our proposed method of RVD-compensation reduced the CV of the RNFL by up to 20%, instead of 11% in the previous model, Contrary to this—and also expectedly—the mean TSNIT profile of RNFL remained unchanged. This demonstrates that our approach was successful in reducing the range of normative data without changing the basic characteristic of the TSNIT curve.

Clinically, when using RNFL measurement for diagnostic purposes,[7–15] the interindividual variation is primarily a problem for the most atypical cases, the outliers of the distribution in the population. We thus looked at the expected difference in RNFL between subjects with large or small RVD (90% to 10% percentiles). This difference amounted to more than 30 µm for large parts of the circumference and reached 50 µm in some areas. When compared to measurements taken at the OD margin, the expected difference increased by 20 microns in large parts of the TSNIT graph.

These new results confirm the importance of considering retinal vessel patterns when analyzing RNFL thickness values. An accurate determination of vessel density is crucial to understand major characteristics of circumpapillary RNFL thickness, such as peak deviation. It is conceivable that a subject with a high RVD in a given area of the TSNIT graph and an incipient RNFL defect just at that location might show up as normal. On the other hand, a healthy subject with a low RVD in some areas might be erroneously classified as pathologic. This could simply be due to the lack of compensation for the RVD influence on RNFL thickness measurements. While the glaucoma expert may well recognize the atypical RNFL distribution and take it into account, this might not hold true for the majority of ophthalmologists using RNFL measurements in routine setting. Taking into account RVD influence on RNFL profile might better discriminate between healthy and pathological cases.

The limitations in this study have been delineated before.[19] So far, our method is based on subjective grading of the vessel diameters, which might introduce a bias. To overcome this, our method will be validated in an independent set of data. Moreover, it should be investigated whether the consideration of physiological factors, such as fovea position, refractive error and optic disc size.[20], influences our results.

Our study was based on the assumption that there is an association between both retinal blood vessels and RNFL distributions. We are interested in developing a method to partially compensate for the intersubject variance. The physiologic basis for this assumption can be found in central nervous system development during fetal formation. This justification has been previously explored.[19,21–23] Given the biological factors, one might expect that regions of higher RVD, with a more developed vessel network, would mark regions of thicker RNFL at birth and through lifetime, when normal circumstances are preserved.

In our opinion, the improved correlations of the current compared to the old model result from the fact that the retinal vessels, especially in the temporal superior and inferior regions, do not assume a radial path, but a curved path deflected towards the macula. This is illustrated in the location of the RVD peaks in Fig. 1, where its maxima, as measured at 3.46 mm, are temporally shifted 10 to 15 degree, when compared to the measurements at the OD margin. It appears thus natural that measuring the RVD at the same location as the RNFL, as in our present model, results in a better correlation between these two parameters.

In conclusion, our proposed method of RVD-compensation of RNFL measurements, both measured at a 3.46 mm diameter circle, presents a clinically relevant relative reduction of variance of up to 20%. Importantly, this work will help to develop more individualized normative values for RNFL measurements, which may in future lead to improvement in early diagnosis of glaucoma.
